# Computed Tomography utilization in minor head injury

**DOI:** 10.1186/1865-1380-7-S1-O4

**Published:** 2014-07-25

**Authors:** Anitha Durai, Rama Prakasha Saya

**Affiliations:** 1 Department of Emergency Medicine, Jawaharlal Institute of Postgraduate Medical Education and Research, Puducherry, India

## Objective

To study the utility of Computed Tomography (CT) in patients with Minor Head Injury (MHI) with respect to certain clinical findings.

## Methods

This descriptive, observational study was conducted at JIPMER, Puducherry, India. All cases of Minor Head Injury (MHI) with a Glasgow Coma Scale (GCS) score of ≥13 who attended the Emergency Department (ED) during the period of 9th September to 30th September were included and the results were analysed using SPSS version 16.

## Results

Of the 132 cases referred for CT brain, 109 had a GCS score of less than 13 on initial evaluation. Among 109 cases with MHI, 90 were males and 64 (58.7%) were in the age group of 14-44 years. 78 cases were Road Traffic Accident victims, 17 were assaulted and 14 had history of fall. Twenty six (23.9%) had abnormal CT findings. Skull fracture was the commonest finding (19,17.4%), followed by contusion (4, 3.7%) and haemorrhage (EDH/SDH/ICH) (3, 2.8%). The logistic regression analysis showed that Loss of Consciousness (LOC) or amnesia (p =0.045) and female sex (p =0.048) were associated with abnormal CT findings.

## Limitations

Single centre study, lack of assessment of all associated variables and limited sample size were the main limitations of the study.

## Conclusion

A higher proportion of abnormal CT scans related to trauma after minor head injury in this study highlights the need for promotion of safety measures in such risk groups. Abnormal CT scans related to trauma after MHI can be predicted by the presence of certain risk factors for the same.

